# Editorial: Chromosomal fragile sites, genome instability and human diseases

**DOI:** 10.3389/fgene.2022.1119532

**Published:** 2023-01-10

**Authors:** Qing Hu, Advaitha Madireddy, Stefano Gnan, Chun-Long Chen

**Affiliations:** ^1^ Department of Pathology, University of Texas Southwestern Medical Center, Dallas, TX, United States; ^2^ Rutgers Cancer Institute of New Jersey, New Brunswick, NJ, United States; ^3^ Institut Curie, Dynamics of Genetic Information, CNRS UMR3244, PSL Research University, Sorbonne Université, Paris, France

**Keywords:** fragile sites, replication stress, genome instability, human disease, DNA damage repair

Chromosomal fragile sites are specific regions of the human genome that are normally stable, but exhibit breaks or gaps on metaphase chromosomes under conditions of stress. As an important source of genome instability, fragile sites are associated with human diseases such as cancer and mental retardation. In this Research Topic, we collected nine contributions from different perspectives, which cover the features and causes of fragile sites, the characterization of fragile sites, as well as the cellular mechanisms at play to maintain their stability.

Based on the frequency of fragility, fragile sites are generally categorized as common fragile sites (CFSs) and rare fragile sites (RFSs). Lokanga et al. summarized recent work about CFSs and RFSs and discussed the similarities and differences between them. CFSs are an intrinsic part of normal chromosome structures and are present in all individuals. Moreover, CFSs are appeared to be conserved throughout mammalian evolution, for example, human FHIT/FRA3B and mouse Fhit/Fra14A2 are orthologs. RFSs are found in a minority of the human population and are inherited in a Mendelian manner. They are often associated with the expansion of repeat elements. RFSs can be further categorized into two groups: folate-sensitive RFSs that are highly sensitive to folate deficiency, and non-folate-sensitive RFSs that are induced by distamycin A or bromodeoxyuridine (BrdU). As the feature of folate-sensitive RFSs, CGG trinucleotide repeats (TNRs) could form secondary structures and perturb DNA replication, thus contributing to their fragility. Garribba et al. tested whether folate deficiency could cause instability at other genomic regions containing CG-rich repeat sequences and showed that a region at Chr2p11.2 displayed an unusual conformation under folate deprivation, leading to the mis-segregation of this locus and Chr2 Aneuploidy.

Although human CFSs have been primarily mapped in lymphocytes and fibroblasts, it has been shown that different CFSs are expressed in different cell types at various frequencies. Characterization of CFSs in other cell types and investigation of CFS expression in different genetic backgrounds are, therefore, important for us to understand the basis of their fragility. In this Research Topic, Balzano et al. assessed CFS expression in human glioblastoma cell lines and found two CFSs that are specific to glioblastoma. They also showed that the fragility of these sites is related to impaired DNA replication. As the major cause underlying CFS fragility, replication stress can arise from various sources, such as oncogene activation, nucleotide depletion, and Transcription-Replication Conflicts (TRCs). A large majority of the highly expressed CFSs host large genes spanning over megabases and transcription-associated replication stress has been proposed as the prominent mechanisms leading to fragility at these large genes. Transcription can either suppress replication initiation to generate large regions that are poor in replication initiation or can generate direct collision with DNA replication machinery. Wu et al. reviewed recent findings on how conflicts during transcription and replication affect chromosome fragility. This mechanism is also supported by the recent study of Munk et al., who deleted 80 kb intron sequences of an extremely large gene *PRKN* and showed that this deletion resulted in a twofold reduction of the PRKN fragility without affecting its expression. The mechanism underlying CFS fragility is not restricted to TRCs. Other features of CFSs such as late replication timing, DNA secondary structure formation, and chromatin modification, can also lead to replication stress and contribute to their fragility. Kodali et al. compared the epigenomic signatures associated with spontaneous and replication stress-induced DNA double-strand breaks (DSBs) and demonstrated the correlation of aphidicolin-induced DSBs with histone 3 lysine 36 trimethylation, which is a marker for active transcription. More interestingly, their result suggested that DSBs were not enriched in the CFS core sequences and rather demarcated the CFS core region. Their analysis suggested that altered replication dynamics are responsible for CFS formation under a relatively higher level of replication stress.

Cellular pathways and signaling components that are involved in mitigating replication stress are important to maintain genome stability. In response to replication stress, the stalled replication forks require stabilization and remodeling to facilitate fork restart. Many proteins that participate in these processes have been shown to affect CFS expression, such as ATR, DNA-PKcs, and some DNA helicases. The function of DNA-PKcs in DNA replication stress is reviewed by Yue et al. The Bloom syndrome DNA helicase BLM belongs to the RECQ family and has been shown to participate in fork restart. Here, Ellis et al. showed that RNF4 facilitates replication fork recovery by regulating BLM. Stalled forks that failed to restart will collapse and use the DNA damage repair (DDR) pathway to recover. Niazi et al. identified 14 DNA repair genes that are associated with chromosomal aberrations, further emphasizing the importance of DDR in promoting genome stability. Deficiencies in DNA damage mechanisms are, therefore, an important source of genome instability and render cells more sensitive to replication stress.

Our understanding of the mechanisms governing chromosomal fragile sites is constantly evolving. In addition to clearly defining the current state of the field, the articles included in this Research Topic present valuable novel insights into the determinants of chromosomal fragility, which contribute to many human diseases.

